# Raman spectroscopy-based identification of nosocomial outbreaks of the clonal bacterium *Escherichia coli*

**DOI:** 10.1007/s10096-015-2511-x

**Published:** 2015-11-12

**Authors:** J. G. Kusters, W. B. van Leeuwen, K. Maquelin, H. E. M. Blok, H. F. M. Willemse, L. A. M. de Graaf-Miltenburg, A. C. Fluit, A. Troelstra

**Affiliations:** Department of Medical Microbiology, University Medical Center Utrecht, Heidelberglaan 100, 3584 CX Utrecht, The Netherlands; Department of Innovative Molecular Diagnostics, University of Applied Sciences Leiden, Leiden, The Netherlands; Center for Optical Diagnostics and Therapy, Department of Dermatology, Erasmus University Medical Center, Rotterdam, The Netherlands; Department of Medical Microbiology and Infectious Diseases, Erasmus University Medical Center, Rotterdam, The Netherlands

## Abstract

DNA-based techniques are frequently used to confirm the relatedness of putative outbreak isolates. These techniques often lack the discriminatory power when analyzing closely related microbes such as *E. coli*. Here the value of Raman spectroscopy as a typing tool for *E. coli* in a clinical setting was retrospectively evaluated.

## Introduction

The control of outbreaks is essential both in hospitals and other healthcare practices as well as in the community. Frequently, molecular typing methods are used to establish the clonal relationships between isolates and confirm the clinical or epidemiological data, thus supporting the decisions in an outbreak situation. A suitable typing technique should have complete typability, be timely, cost-effective, reproducible, and have the correct discriminatory power [1, 2]. In a putative outbreak situation the latter is a crucial determinant of a typing method because over-discrimination will result in missed relationships while under-discrimination results in clustering of potentially unrelated strains and thus unnecessary interventions. However, some bacterial species are highly clonal and most routine typing techniques lack the discriminatory power to allow for reliable typing [1, 2], for example, the molecular typing of *Escherichia coli* is for this reason notoriously difficult.

Raman spectroscopy of bacterial samples is usually performed by a modified light microscopic device. The sample consists of a dried suspension from a bacterial culture and is illuminated with laser light. This will generate a Raman signal that can easily be separated from the laser light by an optical filter that only allows the Raman spectra to pass. These filtered signals are captured by a simple camera device and images are usually stored and analyzed on a small personal computer. As Raman spectra of bacteria are representations of their overall molecular composition (both nucleic and fatty acids, proteins, and carbohydrates) they can be used as highly specific spectroscopic fingerprints of the total cell content. Since Raman spectroscopy typing is based on the analysis of the total bacterial composition (and not only part of its DNA composition), it may therefore provide sufficient discriminatory capability for typing of closely-related microbes such as *E. coli*. However, a systematic review of literature only showed results for typing of *E. coli* using Raman spectroscopy in a preliminary retrospective study in a research-based setting [3].

## Methods

Here the value of Raman spectroscopy as a typing tool for *E. coli* in a clinical setting was retrospectively evaluated. A set of *E. coli* isolates from a putative outbreak setting in a neonatal intensive care unit (NICU) in the University Hospital Utrecht was chosen for this evaluation. Based on epidemiological data and antibiograms four potential outbreaks were defined. Although next generation sequencing (NGS) can probably provide the necessary resolution for the typing of *E. coli*, and it is often referred to as a fast and cost-effective method, in a routine setting Raman spectroscopy is cheaper, faster, and easier to perform [3]. It does not require the complicated sample preparation steps that are needed for NGS nor does it require special DNA/bioinformatics skills and facilities. The data analysis of Raman spectroscopy can be performed in minutes for Raman vs hours for NGS, and data analysis of Raman spectra is performed in a semi-automated almost real-time fashion while NGS usually requires manual input from someone with a bioinformatics background and may take days to complete. The running costs of Raman are significantly less as they are limited to a simple sample carrier vs NGS that requires costly DNA isolation and sequencing kits.

The University Medical Center Utrecht (UMCU) is a tertiary care university hospital, in which the NICU consists of 55 beds divided in 28 intensive care beds on three wards: ten beds on a high care unit (HC) and 17 beds in a medium care unit. Furthermore, six isolation rooms with an anteroom are present.

For all children admitted to the NICU, inventory cultures are taken upon admission and twice weekly afterwards throat and rectum swabs were taken during their subsequent stay on the ward in order to be informed about their bacterial colonization status. In addition to these routine cultures additional clinical specimens were obtained for culture in case of suspicion of infection. All aminoglycoside-resistant *Enterobacteriaceae* isolates were identified to the species level using MALDI-TOF MS, and antibiotic susceptibility testing was performed. In addition, antibiotic resistance surveillance screening among all children present on the ward was performed on a monthly basis. A swab from the patient was obtained and material on the swab was resuspended in liquid broth containing 8 mg/L tobramycin. If growth was observed after overnight incubation at 37 °C, a 20-µl aliquot of the culture was plated on a McConkey agar plate with 8 mg/L tobramycin and a second aliquot was plated on McConkey agar plate with 8 mg/L gentamicin. This combination of this liquid medium and these selective plates only allow the growth of aminoglycoside-resistant isolates and were used to selectively screen for resistant gram-negative isolates. This monthly point-prevalence surveillance is routinely preformed to provide data with regard to resistance development on the neonatal ward and enables the detection of possible outbreaks at an early stage.

In our hospital a surveillance algorithm is used that is based on these point-prevalence surveillance studies, and compares the number of *Enterobacteriaceae* isolates cultured from patients in each ward per month to the historic data. In June 2010 a striking increase in the number of aminoglycoside-resistant *E. coli* isolates was noticed in surveillance cultures of patients residing in the NICU. In the years before this increase very few (i.e. zero to one, or very rarely two) positive patients per month were found. In July 2010 seven positive patients were detected: six in August and five in September. After that, it normalized to the old levels (i.e. zero to one, or very rarely two positive patients per month). These data indicated a potential outbreak running from June to September 2010.

In total 34 isolates (isolated between May 2010 and May 2011) were included in this study: 27 aminoglycoside-resistant *E. coli* isolates from 23 patients and seven control isolates (Table 1). Amongst these were 14 strains isolated from 12 of the 18 patients that had resided in the ICU during this putative outbreak period (from four patients viable aminoglycoside-resistant *E. coli* isolates were no longer available). In addition, epidemiological data (unit, ward, admission and discharge date) were collected. No changes in the procedures at the microbiological laboratory and the strategies for screening, culturing or antibiotic treatment were made prior or during the study period.

**Table 1:**
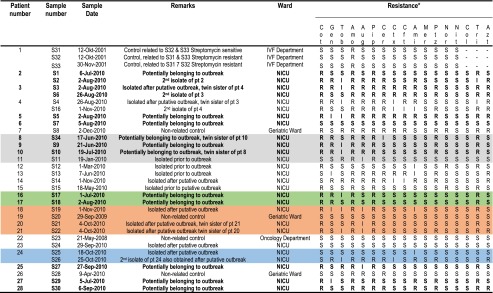
Patients and isolate characteristics

*Breakpoints according to European Committee on Antimicrobial Susceptibility Testing Version 2.0. R = resistant, I = intermediate, S = sensitive, − = Not determined. . Cot: Cotrimoxazole, Gen: Gentamicin, Tob: Tobramycin, Amo: Amoxicilin, Aug: Amoxicilin/Clavulanate, Pip: piperacilin, Cer: Cefuroxime, Ctx: Ceftriaxone, Cft: Ceftazidime, Ami: Amikacin, Mer: Meropenem, Ptz: Piperacillin/Tazobactam, Nor: Norfloxacin, Nit Nitrofurantoïn, Col: Colistin, Tri: trimethoprim, Azt: Azthreonam. In bold are isolates from the patients that resided in the ICU ward during the putative outbreak period. Colored bars represent isolates that are closely related based upon their Raman spectra. Bold lettering represents isolates potentially belonging to the outbreak

**Fig. 1:**
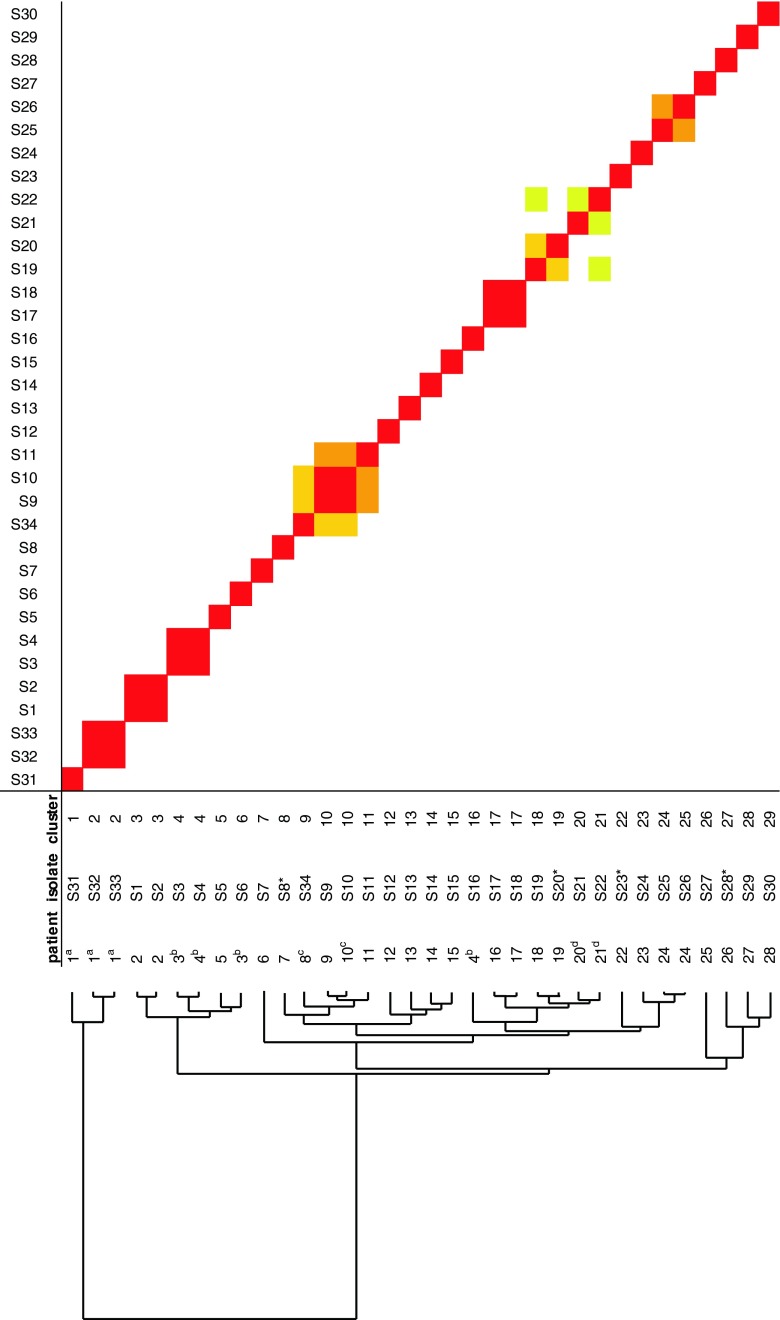
Cluster analysis using the dedicated SpectraCell*RA*® software. Cluster analysis of the spectra was performed using the pairwise similarities (or R2-values) as a distance matrix. These R2-values are visualized in a dendrogram (*left-hand side*) and a correlation matrix table (*right-hand side*). Each horizontal line in the matrix table represents all R2-values of an isolate with all other isolates in the matrix. Strain relatedness was graphically depicted in a dendrogram plot in which each node represented the lowest correlation coefficient (or similarity) between all isolates combined in the cluster defined by this node. The horizontal axis depicts arbitrary units that are specific for this dataset and thus cannot be directly compared with other dendrogram plots obtained from different sets of isolates. By sorting these correlation coefficients based on height, those isolates with high similarity are grouped together and the color indicates the relatedness between isolates. The *red clusters* indicate isolates that have a similarity ≥95 %. Based on previously set thresholds [3] these isolates are classified as indistinguishable. The isolates with a similarity ≥85 to < 90 % are indicated by *yellow*, and those ≥90 % to < 95 % by *orange*. The latter two groups represent clearly distinguishable isolates that have however a high probability to be related. The *white areas* indicate isolates that have a similarity below 85 % and are classified as non-related ^a^ Epidemiologically related control isolates from semen of a single patient ^b, c, d^ Twins; * non-related control isolates

Culturing and sample preparation for typing were performed as described previously [3, 4], and Raman spectra were collected using the SpectraCell*RA*® Bacterial Strain Analyzer (River Diagnostics BV, the Netherlands) according to the manufacturer’s instructions. Cluster analysis using the dedicated SpectraCell*RA*® software version 1.8.1 was displayed as a two-dimensional plot and a dendrogram plot (Fig. 1).

## Results

Among the 27 patient isolates there were 13 isolates that had unrelated Raman spectra. Eight patient isolates formed four clusters (cluster 3, 4, 10, 17; each consisting of two isolates), four isolates formed two potential clusters (cluster 20/21 and 24/25) and the remaining two isolates (S34 and S11) showed profiles related to the profiles of two isolates in cluster 10 (cluster 9/10/11; Fig. 1).

Cluster 3 and potential cluster 24/25 consisted each of two isolates from one patient. Cluster 4 and the potential cluster 20/21 were formed by isolates from two sets of twins. In addition, two of the isolates in the potential cluster 9/10/11 were also obtained from twins. The Raman spectroscopy profiles indicated that only this cluster and cluster 17 are caused by putative transfer of isolates between patients and/or a common source. Epidemiological data show that the two patients involved in cluster 17 were present on the same ward of the NICU at the same time. Three of the four patients involved in cluster 9/10/11, two of them twins, were present on the same ward of NICU at the same time, but patient 11 (isolate S11) was discharged more than five months prior to the three other patients in this potential cluster left the hospital. The cluster therefore consisted of three patients. As epidemiological data do not support a link between patient 11 and the other two isolates from cluster 9/10/11 we believe that this relatedness is most likely a reflection of the known limitation of typing techniques in epidemiological studies. Typing can only provide evidence for unrelated strains not belonging to an outbreak and are unable to show that isolates considered related by typing are epidemiologically related. This means typing methods can only be used to support epidemiological data and should/can never be used to prove a linkage by themselves. Thus it is concluded that the typing data show that the observed increase in aminoglycoside-resistant *E. coli* involved only two small clusters of isolates.

Three epidemiologically linked isolates (S31-S33) that were previously shown to be related by RAPD typing were included in our test panel as related controls. As epidemiologically unrelated control strains, four epidemiologically unrelated aminoglycoside-resistant *E. coli* isolates (S23, S20, S28 and S8) were included. These unrelated isolates were obtained from unrelated patients on the oncology (S23) and geriatric wards (S8, S20, S28), which have no connection with the NICU and were collected well separated in time (May 2008, September 2009, April 2010 and December 2010, respectively). The three epidemiologically related isolates were from a couple that repeatedly underwent IVF-procedures that were complicated by infections of the fertilized ovum and were included as related controls and cultured from two semen samples (S31 and S33) and culture medium used for the procedure (S32), respectively. These three isolates were tested by RAPD and found to have the same RAPD type, but while isolate S31 was Streptomycin resistant, isolates S32 and S33 were Streptomycin resistant (Table 1). The four unrelated control isolates (S3, S8, S20, and S23) were well-separated from each other in the dendrogram plot (Fig. 1). However, isolate S20 showed similarity to NICU isolate S19 and based on the Raman profiles a relationship could not be ruled out, but epidemiological data clearly showed that the isolates are not related. These data indicate that Raman spectroscopy is able to distinguish unrelated *E. coli* isolates. The data showed that isolates S32 and S33 are indistinguishable, whereas the other isolate clustered separately, although the isolates from this patient were more closely related to each other than to the isolates of NICU (Fig. 1). Note however that any typing method can only be used to establish whether isolates are different within the given criteria, and thus cannot be epidemiologically linked. However it is impossible to use them to prove that isolates must be epidemiologically related as they cannot be discriminated with the method. Our data suggest that Raman spectroscopy may have a higher discriminatory power than RAPD typing as it does separate S31 from isolates S32/S33 while still maintaining some relatedness. This may reflect slow divergence of individual isolates from an initially infecting single stain, as is suggested by the antibiotic resistance data with isolate S31 being sensitive and S32/S33 being resistant to streptomycin (Table 1). An alternative explanation is that different strains were selected as it is known that patients can be infected by multiple strains of the same species and in most cases only a single colony from each suspect patient was available for testing.

## Conclusion

While NGS is a promising technique, currently it is still a relatively slow and costly method, especially when single isolates that are suspected to belong to an outbreak have to be typed, whereas Raman spectroscopy provides cheap real-time typing of isolates. It can be concluded that Raman spectroscopy is able to cluster isolates that are suspected to be related based on epidemiological data and their antibiotic profiles. Without typing data and only based on available epidemiological data and antibiograms the isolates would have been grouped into four clusters (Table 1), but typing information proved to be very useful for the analysis of these potential outbreaks as several patients could be excluded from the outbreaks. However, these data need further confirmation before the method can be adapted for routine typing.
